# Effects of *Passiflora edulis* Leaf Extract on Lipid Accumulation in HepG2 Cells: In Vitro Evidence and Molecular Docking Analysis Involving PPARα and SREBP-1

**DOI:** 10.3390/ijms27073003

**Published:** 2026-03-26

**Authors:** Johanny Aguillón Osma, John Sebastián León Villarreal, Nelsy Loango Chamorro

**Affiliations:** 1 Research Group in Basic and Educational Sciences, Faculty of Basic Sciences and Tecnologies, University of Quindío, Armenia 630004, Colombia; neloango@uniquindio.edu.co; 2 Cardiovascular and Metabolic Diseases Biochemistry Research Group, Faculty of Health Sciences, University of Quindío, Armenia 630001, Colombia; jsleonv@uqvirtual.edu.co

**Keywords:** NAFLD, PPARα, SREBP-1c, lipid metabolism, molecular docking

## Abstract

Non-alcoholic fatty liver disease (NAFLD) is characterized by excessive hepatic lipid accumulation and represents a major global health concern. *Passiflora edulis* contains numerous phytochemicals associated with diverse biological activities, including hepatoprotective and hypolipidemic effects. This study evaluated the effects of the ethanolic extract of *P. edulis* leaves on lipid accumulation in a cellular model of NAFLD, as well as its potential effect on transcriptional factors involved in lipid metabolism. HepG2 cells were exposed to steatogenic conditions and treated with the extract at non-cytotoxic concentrations, quantifying intracellular and extracellular triglycerides and cholesterol levels. Additionally, molecular docking analyses were performed to evaluate the interaction of reported *P. edulis* phytochemicals with PPARα and SREBP-1. The results revealed a significant reduction in intracellular lipid content compared to untreated cells, while molecular docking predicted favorable binding interactions between the bioactive compounds in the extract, with higher predicted affinity for PPARα (agonist-like interaction) than for SREBP-1c (antagonist-like interaction). These findings suggest that compounds from *P. edulis* leaves reduce lipid accumulation in liver cells and provide preliminary evidence supporting possible interactions with lipid-regulating transcription factors.

## 1. Introduction

Non-alcoholic fatty liver disease (NAFLD) is recognized as a hepatic manifestation of metabolic syndrome, and its prevalence has been estimated at between 25% and 30% in adults worldwide [1]. It is related to a spectrum of liver diseases ranging from hepatic steatosis to steatohepatitis, fibrosis, and cirrhosis [2]. A diet rich in sugars and saturated fats, as well as a lack of physical activity, are considered to negatively affect health and the development of NAFLD. However, the use of medicinal plants has come to play a vital role in the prevention and treatment of this disease. In this regard, whole plants or parts of them can be used for medicinal purposes; according to the World Health Organization (WHO), more than 80% of the world’s population regularly uses traditional medicines to meet their primary health care needs [3]. Furthermore, more than 50% of new medicines developed and approved for marketing are derived from modified products or active components of medicinal plants [4].

Furthermore, bioactive compounds such as flavonoids, saponins, tannins, and alkaloids have been the subject of recent research due to their recognized antioxidant and hepatoprotective activity [5]. Furthermore, epidemiological studies have highlighted that these bioactive molecules can modulate metabolic pathways involved in inflammatory processes and lipid metabolism [6]. In particular, in addition to their antioxidant capacity, carotenoids interfere with intestinal cholesterol absorption, reducing its plasma levels, which supports their usefulness in the prevention of dyslipidemia [7].

An important factor in the early onset of NAFLD is the modulatory role played by the peroxisome proliferator-activated receptor alpha (PPAR-α). PPAR-α belongs to the nuclear receptor transcription factor family, which includes three isoforms: PPARα, PPARβ/δ, and PPARγ. Among them, PPARα is particularly abundant in the liver but is expressed and active in many other tissues, including skeletal muscle, adipose tissue, and the intestine, kidneys, and heart, which contribute to fatty acid homeostasis [8,9] as the main regulator of lipid metabolism by regulating numerous genes involved in the uptake and activation of fatty acids, peroxisomal and mitochondrial fatty acid oxidation, ketogenesis, triglyceride turnover, lipid droplet biology, gluconeogenesis, and bile synthesis/secretion [10]. In addition, it has other implications, such as glucose metabolism and homeostasis, glycerol control for gluconeogenesis, proinflammatory and anti-inflammatory activity, and oxidative stress [11]. On the other hand, sterol regulatory element-binding protein (SREBP) regulates the expression of enzymes that catalyze the synthesis of fatty acids, cholesterol, triglycerides, and phospholipids [12]. SREBP1c, one of three members of the SREBP family of helix–loop–helix transcription factors, is essential for the genomic actions of insulin in carbohydrate and lipid metabolism and plays a central role in NAFLD progression by promoting the biosynthesis of triglycerides, fatty acids, and cholesterol through its target effectors, which include stearoyl-CoA desaturase 1 (SCD1), fatty acid synthase (FAS), and acetyl-CoA carboxylase (ACC) [13].

Ethnopharmacological information indicates that the Passiflora genus has been used to treat various conditions such as Alzheimer’s disease, cancer, Parkinson’s disease, and liver disease [14]. It has anti-inflammatory, antidiabetic, and sedative properties that have been used for anxiolytic purposes [15,16]. *P. foetida* leaf extract was found to exhibit anticancer activity in colorectal cancer cell models, as well as showing intracellular lipid-lowering effects, suggesting its usefulness in metabolic disorders such as NAFLD [17]. *Passiflora incarnata* leaf extract has been used to treat dyslipidemia, hypertrophy, and hepatic oxidative stress. It has also been shown to reduce the effects of a high-fat diet by lowering TC and TG levels while increasing high-density lipoprotein levels [18].

In particular, *Passiflora edulis*, also known as passion fruit, has been evaluated in extracts, fruit juices, and isolated compounds, showing a variety of health effects and biological activities, such as antioxidant, antihypertensive, antitumor, antidiabetic, and hypolipidemic activities [19,20,21]. In addition, passion fruit seed extract prevented NAFLD by improving liver hypertrophy and hepatic histology in mice fed a high-fat diet [22]. For these reasons, the objective of this research was to evaluate the effects of the ethanolic extract of *P. edulis* leaves (EE) on lipid accumulation in an oleic-acid-induced HepG2 cellular model, as well as its potential interactions with lipid-regulating transcription factors.

## 2. Results

### 2.1. Phytochemical Characterization

The yield percentage of the ethanolic extract obtained from *P. edulis* leaves (EE) was 12.7%. In the phytochemical characterization of the EE, phenols were present in higher proportions with 235.7 ± 9.7 mg EAG/g DE, and total alkaloids were present in lower proportions with 8.3 ± 1.1 mg EC/g ES (Table 1).

### 2.2. Effect of Ethanolic Extract from P. edulis Leaves on Cell Viability

**Figure 1:**
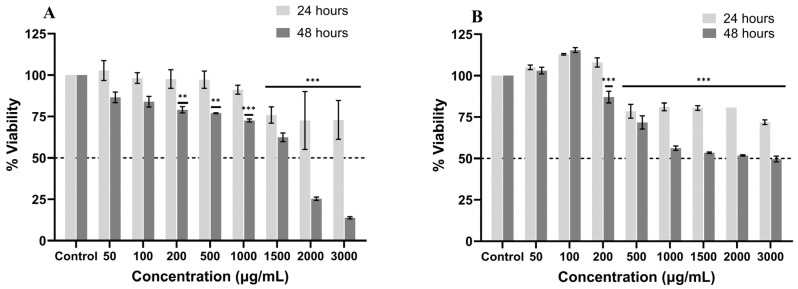
Effect of ethanolic extract of *P. edulis* leaves on viability in HepG2 (**A**) and HFF (**B**) cells at 24 and 48 h. Statistically significant differences between the control and treatment groups: ** *p* < 0.01; *** *p* < 0.001.

The effect on cell viability was evaluated in HepG2 and HFF cells. In HepG2 cells, EE decreased viability; showing a relationship directly proportional to concentration and exposure time; this decrease was statistically significant (*p* < 0.001) for treatments above 1500 µg/mL (75.9%). Likewise, at 48 h, the decrease in viability was significant for treatments above 200 µg/mL (79%), with the 3000 µg/mL treatment (13.86%) showing the greatest reduction in viability in HepG2 cells (Figure 1A). The IC_50_ determined for the viability of HepG2 cells exposed to EE at 24 h was 6663 µg/mL and 1311 µg/mL for 48 h.

EE treatments of HFF cells showed that, after 24 h of treatment, viability decreased in a concentration-dependent manner, which was statistically significant for treatments above 500 µg/mL. However, above this concentration, the decrease in viability was not affected by further increases in concentration. On the other hand, at 48 h, the decrease in viability percentage was significant for treatments above 200 µg/mL and stabilized at 1000 µg/mL. The estimated IC_50_ at both 24 and 48 h was 6716 and 1914 µg/mL, respectively (Figure 1B). When calculating the selectivity index, values of 1.01 and 1.46 were obtained for the 24- and 48-h treatments, respectively. It can therefore be observed that the extract at 24 h of treatment does not have a differential effect between healthy and cancerous cells; however, at 48 h, HepG2 cells showed greater sensitivity to the extract than healthy cells.

### 2.3. Effect of Ethanolic Extract from P. edulis Leaves on Intracellular and Extracellular Triglycerides

The effect of EE on total lipid accumulation in AO-induced HepG2 cells was monitored intracellularly and extracellularly at two incubation times corresponding to 24 and 48 h.

**Figure 2:**
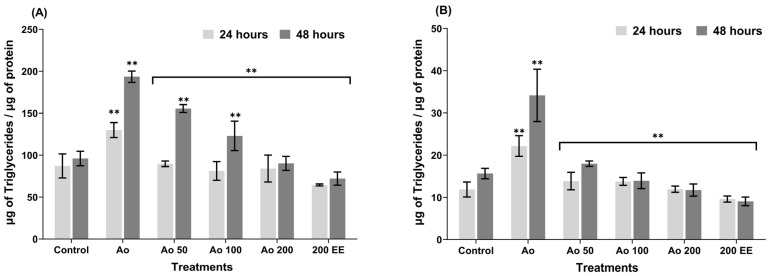
Effect of ethanolic extract of *P. edulis* leaves on the total triglyceride content of both intracellular (**A**) and extracellular (**B**) HepG2 cells in response to 24 and 48 h incubation. Control; untreated cells; Ao, cells with oleic acid (100 µM); Ao50, oleic acid and ethanolic leaf extract 50 µg/mL; Ao100, oleic acid and ethanolic leaf extract 100 µg/mL; Ao200, oleic acid and ethanolic leaf extract 200 µg/mL; 200 EE, ethanolic leaf extract 200 µg/mL alone. ** *p* < 0.01.

As for intracellular TG, during the 24 h incubation period, a statistically significant increase in TG concentration was observed in cells treated with AO (129.83 µg triglycerides/µg protein) compared to the concentration in control cells (87.10 µg triglycerides/µg protein); meanwhile, in the treatments (cells exposed to AO and EE), a statistically significant reduction in TG concentration was observed, showing that all treatments maintain values similar to the control cells. Likewise, it was observed that cells treated only with the extract (200 µg/mL) and without AO showed lower TG values than the control cells, indicating that *P. edulis* has an effect on lipid metabolism (Figure 2A).

During the 48 h incubation period, there was a statistically significant decrease in TG concentration in cells treated with the extract compared to cells containing only AO, demonstrating once again that *P. edulis* extract has the ability to regulate intracellular lipid accumulation in liver cells. A relevant result is that treatment at 200 µg/mL reduces TG concentration to values similar to those of the control cells (Figure 2A). Therefore, the percentage reduction in intracellular triglyceride accumulation was 37.8% and 53.7% for the 24 and 48 h treatment periods, respectively, indicating that the effect increased significantly over time.

On the other hand, when evaluating the concentrations of extracellular TG or TG released into the culture medium of HepG2 cells treated with EE from *P. edulis* leaves at both 24 and 48 h, a significant decrease in TG released from EE-treated cells was observed compared to cells containing AO, showing that there were no significant differences between the values of the treatments and the control cells, even the cells treated only with EE at 200 µg/mL had lower values compared to the control cells (Figure 2B).

In the 48 h incubation assay, behavior very similar to that observed in the 24 h assay was observed, i.e., there was a statistically significant increase in TG release in AO-treated cells compared to the TG concentration in control cells. In addition, a significant decrease in TG concentration was observed in cells treated with EE compared to cells that only had AO (Figure 2B). Likewise, it was evident that the treatments with EE did not present significant differences compared to the control, and even lower values were recorded compared to the control cells, indicating reduced TG secretion into the extracellular medium. The percentage reduction in extracellular triglycerides was 46.2% for the 24 h experiments and 65.8% for the 48 h experiments, indicating that the extract reduces the amount of TG released by HepG2 cells, and this effect is enhanced with increased exposure time.

### 2.4. Effect of Ethanolic Extract from P. edulis Leaves on Intracellular and Extracellular Cholesterol

**Figure 3:**
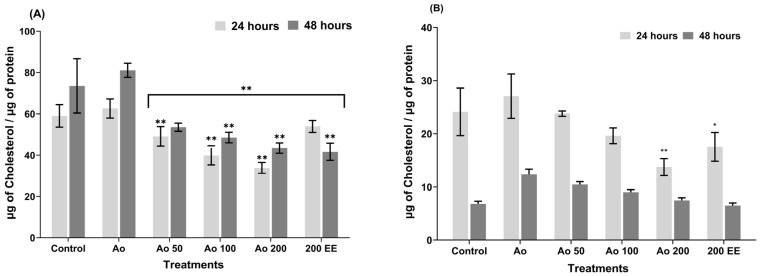
Effect of ethanolic extract of *P. edulis* leaves on the total cholesterol content of both intracellular (**A**) and extracellular (**B**) HepG2 cells in response to 24 and 48 h incubation. Control; untreated cells; Ao, cells with oleic acid (100 µM); Ao50, oleic acid and ethanolic leaf extract 50 µg/mL; Ao100, oleic acid and ethanolic leaf extract 100 µg/mL; Ao200, oleic acid and ethanolic leaf extract 200 µg/mL; 200 EE, ethanolic leaf extract 200 µg/mL alone. * *p* < 0.05; ** *p* < 0.01.

With regard to intracellular cholesterol, during the 24 h exposure period, statistically significant differences were observed in the EE treatments compared to cells containing only AO (Figure 3A). In addition, it was observed that the AO100 and AO200 treatments presented lower cholesterol values than those observed in the control cells, with these differences being statistically significant.

After 48 h of incubation, there was a statistically significant decrease in the cells treated with the extract and the cells that only had AO, showing that all treatments had lower cholesterol values than those observed in the control cells (Figure 3A), indicating that the EE had an effect on intracellular cholesterol metabolism. It is also important to note that cells treated only with EE without AO, both in the 24 and 48 h incubation periods, showed the greatest decrease in cholesterol concentration, indicating that the effect of the extract lasted over time. Finally, the percentage reduction in intracellular cholesterol accumulation for both treatment periods was 46%.

With regard to extracellular cholesterol, during the 24 h incubation period, only the AO200 mixture showed significant differences compared to cells containing only AO (Figure 3B). Similarly, the AO200 mixture generated lower values compared to the control cells. In the assay performed at 48 h, cholesterol levels in cells treated with the mixture of AO and EE from *P. edulis* leaves were close to the levels of cholesterol released into the medium by the control cells (Figure 3B). In addition, the percentage reduction in cholesterol secretion was 49.5% after 24 h of treatment and 40.1% after 48 h of treatment.

### 2.5. Molecular Coupling of the Compounds Present in P. edulis Against SREBP-1 and PPARα

**Figure 4:**
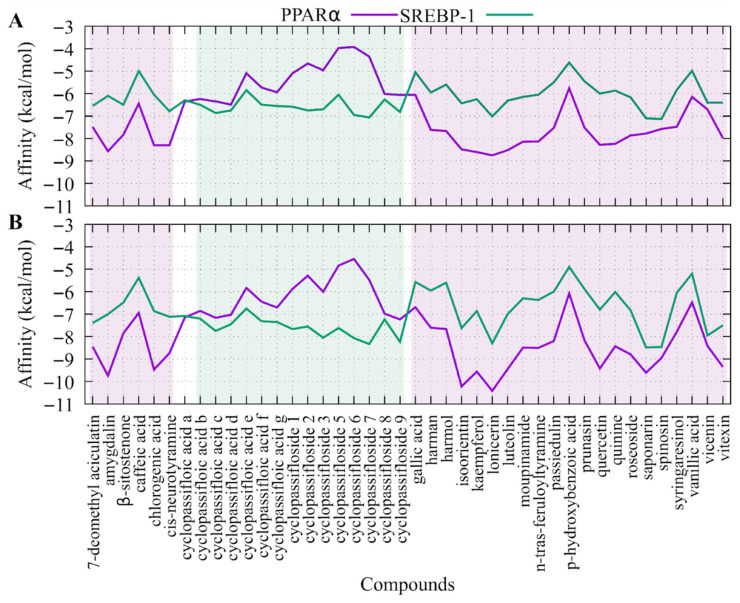
Average binding affinities of each compound for each protein. (**A**) shows the results obtained by Autodock Vina and (**B**) by the Smina software. The compounds that showed the best results for PPARα and SREBP-1 are shaded in purple and green, respectively.

Figure 4 shows the affinity values of each compound for the two proteins of interest (SREBP-1 and PPARα). From the perspective of the average affinity or binding energy for each molecule, PPARα showed affinity with 64.28% of the compounds evaluated, with the compounds isoorientin, amygdalin, lonicerin, kaempferol, and luteolin showing the best affinities. On the other hand, the affinity of the compounds reported in *P. edulis* for SREBP-1 only includes 27.2% of the compounds evaluated, with cyclopassifolic acids and cyclopassiflosides, saponarin, and spinosin being the compounds with the highest affinity for this transcription factor.

In order to determine which compounds would show the best effects in a cell assay, the Rank-by-Rank consensus method was implemented to combine the data from both proteins and both programmes. Table 2 shows the consensus results, with the compounds with the best rankings highlighted in green. In this vein, it would be expected that, in an experiment using the compounds separately, the compounds indicated here would exhibit better biological effects. In general, it can be observed that the compounds with the best rankings are flavonoids and glycosides, such as 7-de-O-methylaciculatin, isoorientin, kaempferol, luteolin, saponarin, spinosin, vitexin and lonicerin, with the latter compound showing the best results for both proteins and both programmes.

## 3. Discussion

The differences found in the phytochemicals present in EE from *P. edulis* leaves may vary depending on the methodologies and standards used; the production and quantity of secondary metabolites vary from one place to another and from one environment to another, which may also have an impact [23]. This could be due to genetic, environmental, and ecological factors, including latitude, longitude, precipitation, climate change, soil microbiome and nutrients, temperature, phytopathogens and cultural practices, post-harvest storage, and processing, among other factors.

The EE of *P. edulis* leaves had a high total phenolic content of 235.7 mg EAG/g DE, compared to that reported by Aguillón et al. [24] and Guimarães & Modolo [25], who reported lower results for this same extract, with total phenolic content of 150.3 mg EAG and 122.0 mg EAG, respectively. However, when considering extracts obtained with other solvents, such as the aqueous extract of *P. edulis* leaves with phenolic contents of 10.45 mg EAG [26], this difference may be due to the different polarities that affect the efficiency of phenol extraction. Regarding the different types of phenols, it has been reported that chlorogenic acid and its derivatives have the ability to decrease TG accumulation in AO-induced HepG2 cells [27]; another study confirmed the protective effect of chlorogenic acid (80 µM and 50 µM) against the formation of lipid droplets induced by free fatty acids (FFA) and the elevation of ROS in HepG2 cells [28]. Chlorogenic acid also improved blood lipid metabolism by alleviating FFA, TG, and modulating the hepatic level of pACC through the AMPK pathway [29]. Additionally, this compound has been reported to increase AMPK and ACC phosphorylation and reduce the expression of SREBP-1C, ACC, and FAS in HepG2 cells [30].

Furthermore, anthocyanins have been found to activate AMPK in human HepG2 cells and can decrease the level of SREBP-1c [31]; in addition, other phenolic compounds that have been found, such as caffeic acid, catechins, and procyanidins, have the ability to improve the AMPK/ACC/SREBP-1c pathway, reducing hepatic steatosis due to FFA overload [32].

Regarding the total flavonoid content, Santos et al. [33] found 29.4 mg EC in the EE of *P. edulis* leaves, which is lower than the results reported in this study. Sotiropoulou et al. [34] mention that quercetin stands out for its hepatoprotective role, where in a study with rats treated with ethanol, quercetin proved to be beneficial in chronic ethanol-induced liver injury due to the increase in glutathione content. The inhibition of glucose uptake and cell proliferation was also evident, finding that quercetin is capable of improving insulin resistance, decreasing fat accumulation and increasing cell proliferation, while inhibiting IL-8 and TNF-α levels, with an increase in cellular glutathione. In addition, luteolin has been found to exhibit anti-inflammatory, antioxidant, antitumor and immune regulatory activity [35], where this flavonoid can significantly reduce body weight and liver index, restoring serum lipid levels, inhibiting hepatic lipid accumulation and improving insulin resistance in Wistar rats.

Regarding the total tannin content, Saravanan and Parimelazhagan [36] report a quantity of 67.90 ± 3.02 mg EAG (gallic acid) in the methanolic extract of *Passiflora ligularis*, which is higher than that reported in our research. De Huang et al. [37] mention that epigallocatechin gallate significantly reduced the development of obesity, hyperglycemia, insulin resistance, inflammatory cytokines, hypercholesterolemia, and fatty liver in rats fed a high-fat diet. On the other hand, Li et al. [38] note that tannic acid decreased the mRNA expression of genes related to lipogenesis and attenuated lipid accumulation in vivo, observing that the characteristics of NAFLD, including body weight, liver mass, fat mass, and serum lipid profile, improved with tannic acid supplementation in vivo.

Finally, with regard to the total alkaloid content, De Sousa et al. [39] mention that the leaves of *P. edulis* contain alkaloids such as harmine, harmalol, harmaline, and harman, a β-carboline alkaloid. Alkaloids have also been detected in extracts and fractions from the bark of *P. edulis*, where different indole alkaloids such as harmine, harmaline, and harmalol have been identified [40]. Various reports have mentioned a wide range of pharmacological activities, including anti-inflammatory [41], antioxidant, and anticancer activities [42,43], making it a possible candidate for NAFLD.

Although HepG2 cells present recognized limitations—such as their origin from hepatocellular carcinoma, altered metabolic regulation compared to primary hepatocytes, and an incomplete representation of hepatic physiological complexity, including xenobiotic metabolism and multicellular liver interactions—they remain one of the most widely used in vitro models for studying lipid accumulation. Their human origin, stable phenotype, high reproducibility, and ease of culture make them particularly suitable for preliminary screening of compounds with potential lipid-lowering activity. Moreover, HepG2 cells retain relevant hepatic metabolic functions, including lipogenesis and lipoprotein secretion, enabling controlled evaluation of intracellular triglyceride and cholesterol modulation [44].

Cell viability assays showed that the EE from *P. edulis* leaves had an antiproliferative effect on HepG2 at 24 and 48 h (IC_50_/24 h = 6663 µg/mL–IC_50_/48 h = 1311 µg/mL). In addition, the maximum inhibition percentage of EE was 27% at 24 h and 86.1% at 48 h. Similarly, EE was found to have a greater cytotoxic effect on HepG2 cells compared to the HFF cell line, indicating greater selectivity for the tumor cell line, which is favorable, as differential antiproliferative activity is sought. Similarly, in the study conducted by Villada et al. [45], they found that cell viability had an effect on SW480 cells, dependent on the dose and exposure time. These effects could be related to the presence of certain metabolites that have been described in extracts of *P. edulis*, such as terpenes and glycosides, which have been found in both aqueous extracts of the fruit and ethanolic extracts of the leaves; these include ‘passiflorine’ and ‘passicapsin,’ alkaloids, tannins, quinones, flavonoids, carotenoids, anthocyanins, ascorbic acid, vitamins B6, vitamin A, riboflavin, and lactones [15], which have been found in different plant structures and may contribute to the biological effects observed in this study.

Regarding other species from the Passiflora genus, it has been reported that *Passiflora ligularis* EE showed significant antiproliferative activity on human hepatocellular carcinoma cells (IC_50_: 50 mg/mL); however, no cytotoxic effect was observed in primary hepatocyte cultures [46]. On the other hand, *Passiflora alata* leaf extract has been reported to have cytotoxic or anticancer activities attributed to its constituent flavonoids and saponins, where the data obtained indicated that *P. alata* inhibits cell proliferation >75% against the four tumor cell lines (PC-3, K-562, HepG2, and S180) using the MTT method, and IC_50_ < 30 µg/mL [47].

On the other hand, the increase in lipid accumulation in HepG2 cells promoted by the presence of oleic acid triggers the generation of high levels of oxygen free radicals compared to control values [48], due to the high rate of lipid peroxidation that is generally associated with damage at the cellular level, which can lead to alterations in cell division controls, causing the cell to develop other pathological conditions, such as steatohepatitis, cirrhosis, or more alarming consequences such as cancer or death. In this study, HepG2 cells were treated with pathophysiological levels of oleic acid to mimic the entry of excess free fatty acids into hepatocytes, leading to steatosis, because hepatocytes activate enzymes associated with lipogenesis, resulting in increased TG synthesis and greater lipid accumulation in the liver [48], a situation that was evident in this research. However, in this study, TG accumulation was substantially reduced both intracellularly (50.34%) and extracellularly (56.65%) at 24 h of incubation and at 48 h of incubation, it was reduced by 62.74% and 48.68%, respectively, compared to cells that were induced to lipid accumulation with AO, suggesting a potential protective role against NAFLD in vitro. Similarly, studies have reported that blackberry (*Rubus* sp.), wild blueberry (*Vaccinium* spp.), strawberry (*Fragaria ananassa*) and chokeberry (*Aronia melanocarpa*) have an inhibitory effect on oleic-acid-induced TG accumulation in HepG2 cells [49]. Furthermore, in a study conducted by Mun et al. [50] using an animal model fed a high-fat diet until the development of fatty liver, the administration of *Curcuma longa* extracts reduced lipid accumulation. Similarly, *Lonicera caerulea* extract has been reported to show a significant decrease in intracellular TG in HepG2 cells that were induced to accumulate lipids with AGL for 24 h [51]. With regard to TC levels, our study also showed a reduction both intracellularly (46.05%) and extracellularly (49.18%) after 24 h of incubation, and, after 48 h of incubation, the reduction was 48.68% and 47.78%, respectively. Similarly, it has been reported that the ethanolic extract of *Liriope platyphylla* root significantly decreased serum TC levels in mice fed a high-fat diet [52].

With regard to the in-silico results of molecular docking, several reports have been published on the biological activity of certain compounds and their interaction with the PPARα and SREBP-1 proteins, such as luteolin. Yin et al. [53] found that treatment with luteolin can significantly inhibit body weight gain in mice and hepatic TG levels. This result is attributed to the fact that luteolin regulates hepatic lipid homeostasis by inhibiting LXR activation, which causes a decrease in lipid accumulation and suppresses LXR-SREBP-1 signaling. Additionally, kaempferol can improve insulin resistance to reduce hepatic fat overaccumulation and prevent the progression of NAFLD, as well as promote hepatic expression of CYP4A1 (cytochrome P450) secondary to direct upregulation of hepatic PPARα expression [54].

In the case of saponarin, it has been reported to inhibit the expression of SREBP-1 and PPARγ [55], which are associated with lipid metabolism, the expression of lipogenic enzymes such as FAS, and TG production. Consequently, saponarin suppresses TG accumulation by stimulating AMPK, which is an important energy regulator. Several natural products, including isoorientin, lonicerin, kaempferol, and luteolin, have shown promising results in preclinical studies through the activation of AMPK and the regulation of its proteins related to lipid metabolism [56,57,58]. AMPK directly inhibits the activation of SREBP-1c, a transcription factor that controls lipid metabolism, with SREBP-1c activating the expression of FAS and SCD-1, two proteins crucial in hepatic fatty acid synthesis, to promote de novo lipogenesis. Tie et al. [59] found that treatment with kaempferol and kaempferide in HepG2 cells reduced the AO-induced increase in SREBP-1c, FAS, and SCD-1, suggesting that these flavonoids inhibit intracellular lipid accumulation by suppressing the expression of lipogenic proteins.

## 4. Materials and Methods

The plant material (*P. edulis* leaves) was collected in the village of Tierra Blanca (Roldanillo, Valle del Cauca, Colombia), located at the global geographic coordinates 4° 24′0″ N, 75.8489° W, at an altitude of 939 m above sea level. The samples were taken to the Biochemistry Laboratory of the Faculty of Health Sciences at the University of Quindío.

*Preparation of the extract*. The leaves of *P. edulis* were washed and then dried in a circulating air oven at a constant temperature of 40 °C. The material was then pulverized and finally leached for 8 days using 96% ethanol, with the leachate being constantly recirculated. The chlorophylls were then separated via liquid–liquid extraction with ethanol–water (1:1) [60]. The ethanol was evaporated at a reduced pressure of 60 mbar and at a temperature below 30 °C in a rotary evaporator (Heidolph, Heidolph Instruments GmbH & Co. KG, Schwabach, Germany). The resulting suspension was subjected to freeze-drying (Telstar LyoQuest, Syntegon Telstar, SLU, Barcelona, Spain) and the product obtained was stored protected from light at −20 °C.

The percentage yield was determined based on the following formula:

% *yield* = (*dry extract weight/dry leaf weight*) × 100%.

*Determination of total phenol content.* This was determined using the Folin Ciocalteu reagent method [61]. An aliquot of 50 µL of the ethanol extract (EE) (1 mg/mL) was taken and 2.5 mL of Folin Ciocalteu reagent (1:10 dilution) and 2 mL of 7.5% (*w*/*v*) sodium carbonate (Na_2_CO_3_) were added, mixed until homogeneous, and incubated at 40 °C for 15 min. A solution of 2 mL of Na_2_CO_3_ in 2 mL of distilled water was used as a blank, and the reading was taken at a wavelength of 765 nm. The results are expressed as mg equivalent of gallic acid per gram of dry extract (mg EAG/g DE).

*Determination of total flavonoid content.* To an aliquot of 150 µL of EE (1 mg/mL), 45 µL of 5% sodium nitrate (NaNO_3_) was added. At five and six minutes, 90 µL of 10% aluminum chloride (AlCl_3_) and 300 µL of 1 M sodium hydroxide (NaOH) were added, respectively. The final volume of the mixture was adjusted with distilled water to 1.5 mL. Finally, the mixture was allowed to stand for 10 min at room temperature and read at a wavelength of 510 nm. The results are expressed as mg catechin equivalents per gram of dry extract (mg CE/g DE) [62].

*Determination of total polysaccharide content*. This was determined using the phenol-sulfuric acid method [63]. To an aliquot of 100 µL of EE (1 mg/mL), 100 µL of 5% phenol and 500 µL of 95% sulfuric acid (H_2_SO_4_) were added, mixed until homogeneous, and incubated at room temperature for 15 min. The reading was taken at a wavelength of 490 nm, and distilled water plus H_2_SO_4_ was used as the blank. The results are expressed as mg glucose equivalents per gram of dry extract (mg GE/g DE).

*Determination of total tannin content.* To an aliquot of 250 µL of EE (1 mg/mL), 500 µL of bovine serum albumin solution in 0.2 M acetic buffer with pH 5.0 (pH adjusted with 0.17 M NaCl) was added, mixed thoroughly, and allowed to stand for 15 min. It was then centrifuged at 5000 rpm for 15 min. The supernatant was discarded and the precipitate was diluted with 1 mL of aqueous solution containing 1% sodium dodecyl sulfate (SDS) and 4% triethanolamine. Finally, 250 µL of 0.01 M ferric chloride (FeCl_3_) in 0.01 M hydrochloric acid (HCL) was added and allowed to stand for 30 min. The reading was taken at a wavelength of 510 nm. As a blank, 1 mL of aqueous solution (1% SDS and 4% triethanolamine) was used in 250 µL of the 0.01 M FeCl3 solution in 0.01 M HCL. The results are expressed as mg equivalent of tannic acid per gram of dry extract (mg EAT/g DE) [62].

*Determination of total alkaloid content.* This was determined using the bromocresol green method [64]. A 1 mL aliquot of EE (1 mg/mL) was taken and 5 mL of phosphate buffer (pH 4.7) and 5 mL of bromocresol green solution were added. Next, 2 mL of chloroform was added and mixed. This last step was repeated two more times. Finally, the extract was collected in a 10 mL volumetric flask and read at a wavelength of 470 nm. The results are expressed as mg quinine equivalents per gram of dry extract (mg EQ/g DE).

*Cell culture.* HepG2 (purchased from ATCC code HB 8065) and HFF (purchased from Cytion, code 305790) cell lines were maintained and propagated in Dulbecco’s Modified Eagle’s Medium (DMEM) (Gibco) with 25 mM glucose and 2 mM L-glutamine, supplemented with 10% fetal bovine serum (FBS) (Gibco), 100 IU/mL penicillin, 100 µg/mL streptomycin (Gibco), and 1% nonessential amino acids (Gibco). They were incubated at 37 °C in a humid atmosphere of 95% air and 5% CO2. The different biological assays were performed while the cells were in the exponential phase and had a confluence of more than 80%.

*Cell viability.* Cell viability assays were performed using the sulforhodamine B (SFB) method [65]. In brief, 10,000 cells/well from each cell line were cultured in 96-well culture plates in a final volume of 200 μL of culture medium and incubated for 24 and 48 h in the presence of EE from *P. edulis* leaves at different concentrations (50–3000 µg/mL, dissolved in DMEM medium). After the exposure period to the extract, 100 μL of cold trichloroacetic acid at a concentration of 15% (*v*/*v*) was added to each well. They were incubated at 4 °C for 1 h in the dark, the acid was removed, and four washes with water were performed. They were then left to dry at room temperature, and finally, 100 μL of SFB (0.4% *w*/*v* diluted in 1% acetic acid) was added per well. They were incubated for 30 min at room temperature, and then excess SRB was removed by washing the adhesion surface with acetic acid (1% *v*/*v* in distilled water). The plates were left to dry at room temperature for 24 h. For absorbance reading, SRB was solubilized by adding 200 μL of Tris-HCl buffer (10 mM pH 10.5) to each well and shaking orbitally for 15 min. The optical density was read at 490 nm by spectrophotometry.

Three biological assays were performed, with each assay repeated three times for each treatment and control. Cell viability was calculated using the following equation:

% *viability* = (*ABt*/*ABc*) × 100

where *ABt* = absorbance of treatments; *AB_C_* = absorbance of untreated cells.

*The Selectivity Index* (SI) of the treatments evaluated was defined as the ratio of cytotoxicity between non-cancerous cells and cancerous cells. Treatments reporting an SI value greater than 3 are considered to have high selectivity. The following equation was used:

*SI* = (*IC*_50_
*in HFF cells*)/(*IC*_50_
*in HepG2 hepatocellular cancer cells*)

*Effect of the extract on total lipids (cholesterol and triglycerides).* Briefly, 10,000 HepG2 cells/well were cultured in sterile 6-well plates in a final volume of 2000 μL of culture medium until 90% confluence was reached. The EE of *P. edulis* (50, 100, and 200 µg/mL non-cytotoxic concentrations) and oleic acid (100 µM) were evaluated. The cells were incubated for 24 and 48 h. The levels of cholesterol and triglycerides were quantified both intracellularly (cell lysate) and extracellularly (culture medium) for each treatment. As a positive control, cells were cultured with oleic acid only; and as a negative control, cells were cultured with culture medium containing the oleic acid solvent (1% albumin in PBS) [66].

Once the exposure period of the extract had elapsed, the culture medium was removed and stored (for extraction and quantification of extracellular lipids). Subsequently, the monolayer was washed with 1X PBS, and the cells were lysed with 1000 µL of lysis buffer (20 mM Hepes, 420 mM NaCl, 1% Triton, 0.5% SDS, pH 7). Immediately, the boxes were incubated at room temperature with constant shaking for 10 min.

*Normalization of lipid concentration.* The assay was performed according to Walker [67]. After cell lysis, 50 µL of the lysate was taken from each of the treatments, controls, and standards, 500 µL of the BCA working reagent (50:1, bicinchoninic acid solution/copper sulfate pentahydrate solution 4%) was added, and, immediately, test tubes were covered and incubated at 37 °C for 30 min. Finally, they were kept at room temperature for 10 min before measurement. The reading was taken at 562 nm. The calibration curve was performed with albumin.

*Lipid extraction and quantification*. Lipid extraction was performed according to the method established by Blight and Dyer [68]. The volume of cell lysate was normalized against the lowest protein concentration observed in either the treatments or controls. A normalized and measured volume of 400 µL of cell lysate was taken for the quantification of the extracted lipids using the commercial Liquicolor^®^ (Human) kit for triglycerides and cholesterol, independently for each one. The same process was followed for the culture medium. Subsequently, 500 µL of chloroform and 1 mL of methanol were added, mixed well, and placed in orbital agitation at 180 rpm for 1 h. Next, 500 µL of chloroform and 500 µL of water were added, forming two phases. It was then centrifuged at 3000 rpm for 3 min to achieve better separation, then the chloroform phase was removed to a new tube, and the chloroform was allowed to evaporate completely overnight. Finally, 500 µL of quantification reagent provided by the Human liquicolor^®^ colorimetric kit was added to analyze either cholesterol or triglycerides, incubated for 10 min, and spectrophotometric reading was performed at a wavelength of 500 nm, also reading the absorbance of the standard provided by the kit.

The results were expressed as µg of lipid/µg of protein for each time point. To convert the absorbances to a concentration unit, the following equation recommended by the commercial Liquicolor^®^ (Human) kit was used:

*Lipid concentration* = 200 × (*ABt*/*ABe*) (mg/dL)

*ABt* = Absorbance of treatments and controls; *ABe* = Absorbance of the standard, either cholesterol or triglyceride

*Molecular docking calculations.* Protein–ligand molecular docking calculations were performed for some components of the extract on the human proteins PPARα and SREBP-1. For this purpose, 10 different structures were used for the PPARα protein (Ensemble docking). The protein structures were obtained from the Protein Data Bank, PDB (https://www.rcsb.org/, accessed on 7 November 2024). For the PPARα protein, the structures with PDB codes 2ZNN [69], 3ET1 [70], 3SP6 [71], 3SP9 [71], 3VI8 [72], 6KB2 [73], 5HYK [74], 1I7G [75], 2NPA [76], and 6KBA [72]. For the SREBP-1 protein, there is only one structure deposited in the PDB with code 1AM9 [77]; however, in this crystal, there are two repetitions of the protein, which were used as two conformations of that protein.

The preparation of the receptors consisted of the removal of water molecules and other co-crystallized ligands, followed by the addition of polar hydrogens and the assignment of Kollman charges. This was done using the Autodock Tools v1.5.6 program [78]. In the case of ligands, a list of 42 compounds present in *P. edulis* leaves was obtained from the Foodb database (https://foodb.ca/, accessed on 12 November 2024). Two programs were used, Autodock Vina [79] and Smina [80]. A total of 3024 molecular docking calculations were performed. All data obtained from this part were analyzed and organized according to the consensus method known as Rank by Rank, using the equation:

$${RbR}_{i}=\frac{1}{n}\sum_{j}r_{i}^{j}$$

where *n* is the total number of programs used (in this case, 2). Using the values obtained for binding affinities as a reference, a value $r_{i}^{j}$ is assigned, which is equivalent to the location of compound *i* with respect to the other compounds. Thus, for example, if a compound occupies position 5 in the list, its score will be 5 for program *j*. Finally, the average of the results of the programs used is obtained.

*Statistical design.* Data are expressed as the mean ± standard deviation of the results performed in triplicate for each independent assay. The spectrophotometric data obtained on the concentration of TG and CT at different stages of treatment were analyzed using a one-way ANOVA to determine the existence of significant differences among the stages. Subsequently, a multiple range test was applied to identify which means differed significantly from each other, were analyzed using GraphPad Prism 8.0 (GraphPad Software Inc., San Diego, CA, USA). A *p* value of <0.05 was considered statistically significant. In cases where the data did not meet the assumptions of normality or homoscedasticity, the non-parametric Kruskal–Wallis test was used.

## 5. Conclusions

The ethanolic extract (EE) of *P. edulis* leaves contains high levels of phenolic compounds, polysaccharides, and flavonoids. Regarding cytotoxicity, EE exhibited differential effects between the two evaluated cell lines, with greater selectivity toward the HepG2 hepatic cell line. Notably, treatment with EE resulted in a statistically significant reduction in both intracellular and extracellular triglyceride (TG) and total cholesterol (TC) levels across the tested concentrations.

In silico molecular docking predicted that several bioactive compounds including isoorientin, lonicerin, kaempferol, luteolin, and amygdalin may interact with the transcription factor PPARα, suggesting a potential association with pathways related to fatty acid oxidation. Likewise, compounds such as lonicerin, saponarin, spinosin, and cyclopassifloside VII showed predicted binding to SREBP-1, which could be hypothetically linked to reduced de novo lipogenesis. However, these interactions are based solely on computational predictions and were not experimentally validated. Overall, these findings expand the current knowledge of *P. edulis* as a promising botanical source with lipid-lowering effects in vitro and support its potential relevance in strategies aimed at mitigating NAFLD, warranting in vivo assays.

## Figures and Tables

**Table 1:** Phytochemical characterization of the EE of *P. edulis*.

Metabolite Type	Total Metabolite Content (mgEQ)
Phenols	235.7 ± 9.7
Polysaccharides	221.9 ± 16.4
Flavonoids	182.4 ± 7.9
Tannins	45.1 ± 3.5
Alkaloids	8.3 ± 1.1

**Table 2:** Final consensus of the results of molecular docking of the phytochemical of *P. edulis* compounds on transcription factors (the highest-ranked compounds are highlighted in green).

Compound	PPARα	SREBP-1	MIX
7deomethylaciculatin	16.70	13	14
Amygdalin	5.19	24	13.5
chlorogenic acid	8.38	27	16.5
cisnfeuroyltyramine	11.57	14	12
cyclopassifloic acid c	27.99	7.5	17
cyclopassifloic acid d	27.18	11.5	18
cyclopassifloside1	35.27	11	23
cyclopassifloside3	32.7	8.5	21
cyclopassifloside6	40.85	5.5	23.5
cyclopassifloside7	39.15	3	22
cyclopassifloside9	27.05	6.5	15.5
Isoorientin	5.05	15	8
Kaempferol	6.85	24	15
Lonicerín	5.4	4.5	3.5
Luteolin	7.05	22	14
Quercetin	8.95	29.5	18.5
Saponarin	10.85	1	4.5
Spinosin	12.9	2	6.5
Vitexin	11.2	18	13.5

## Data Availability

The data presented in this study are available on request from the corresponding author. As the information is part of a degree project, it belongs to the University of Quindío, according to its intellectual property protocol.

## Abbreviations

The following abbreviations are used in this manuscript:

NAFLD	Non-alcoholic fatty liver disease
PPARα	The peroxisome proliferator-activated receptor alpha
SREBP-1	The sterol regulatory element-binding protein type 1
HepG2	Cell line exhibiting epithelial-like morphology that was isolated from a hepatocellular carcinoma
WHO	The World Health Organization
SCD1	Stearoyl-CoA desaturase 1
FAS	Fatty acid synthase
ACC	Acetyl-CoA carboxylase
EE	The ethanolic extract of *P. edulis* leaves
mg EAG/g DE	mg equivalent of gallic acid/g of dry extract
mg EC/g ES	mg equivalent of caffeine/g of dry extract
mgEQ	mg equivalent
HFF	Fibroblast cell that was isolated from the foreskin of a donor
AO	Oleic acid
TG	Triglycerides
TC	Cholesterol
IC50	Medium Inhibitory Concentration
FFA	Free fatty acids
ROS	Reactive oxygen species
AMPK	AMP-activated protein kinase pathway
IL-8	Interleukin 8
TNF-α	Tumor necrosis factor alpha
CYP4A1	Enzyme cytochrome P450 4A1
